# Functional, Rheological, and Sensory Properties of a Whey Protein Phospholipid Concentrate‐Based Ready‐to‐Mix Beverage System

**DOI:** 10.1111/1750-3841.71536

**Published:** 2026-10-09

**Authors:** Fatemeh Jalil Mozhdehi, Sheng‐Luen Shih, Susan G. Larson, Scott A. Rankin, Bradley W. Bolling, Gulustan Ozturk

**Affiliations:** ^1^ Department of Food and Nutritional Sciences University of Wisconsin‐Madison Madison Wisconsin USA; ^2^ Center for Dairy Research University of Wisconsin‐Madison Madison Wisconsin USA; ^3^ Department of Biological Systems Engineering University of Wisconsin‐ Madison Madison Wisconsin USA

**Keywords:** choline, functional properties, rheology, sensory evaluation, whey protein phospholipid concentrate (WPPC)

## Abstract

Whey protein phospholipid concentrate (WPPC) is an underutilized dairy coproduct obtained during whey protein isolate (WPI) production that contains whey proteins and milk fat globule membrane‐derived phospholipids, including phospholipid‐associated choline. These components confer functional benefits; however, data on their physicochemical and sensory performance in ready‐to‐mix (RTM) systems remains limited. A WPPC‐based RTM beverage was formulated using a WPPC powder containing 70.3% protein, 17.6% fat, and 3.22 mg choline/g. The developed RTM powder provided 10 g protein per 100 g prepared beverage when reconstituted with 2% milk. The functional, physicochemical, rheological, and sensory properties of the reconstituted powder were evaluated and compared with a reconstituted commercial WPI‐based RTM powder. Water‐holding capacity, heat stability, apparent emulsifying behavior, apparent dispersity, sedimentation, particle size, and zeta potential were assessed at pH 4.5, 7.0, and native (unbuffered) (6.35 to 6.51). Rheological properties were evaluated at pH 7.0. Consumer liking was assessed using a 9‐point hedonic scale (*n =* 100). The WPPC RTM exhibited pH‐dependent functionality, with greater heat stability, apparent dispersity, and reduced sedimentation at native (unbuffered) and neutral pH compared with pH 4.5 (*p* < 0.05). Although water‐holding capacity and viscosity were lower for the WPPC RTM than for the commercial WPI RTM, stable flow behavior was observed. The commercial WPI beverage received higher liking scores for overall liking, aroma, flavor, aftertaste, and color, and higher purchase intent (*P* < 0.05), while appearance and texture did not differ. Overall, the findings demonstrate the feasibility of incorporating WPPC into an RTM protein beverage formulation.

**Practical Applications:**

This study demonstrates that whey protein phospholipid concentrate (WPPC), a phospholipid‐rich whey coproduct, can be effectively incorporated into ready‐to‐mix (RTM) systems. The results provide formulation‐relevant insights into the functional and physicochemical behavior of WPPC across pH conditions and its sensory performance relative to whey protein isolate‐based beverages. These findings support the use of WPPC as a value‐added ingredient in protein beverages and nutritional supplement formulations.

## 1 Introduction

The growing demand for protein‐enriched and functional beverages has driven continued interest in dairy‐based ingredients that offer both nutritional value and favorable physicochemical properties (Giri et al. 2023). Dairy proteins are typically derived from casein or whey fractions and are widely used in beverage and powder‐based formulations due to their high nutritional quality and functional performance (Etzel 2004). Cheese whey, whey protein concentrate (WPC), and whey protein isolate (WPI) have long been incorporated into protein beverages and ready‐to‐mix (RTM) powder systems (Temelli et al. 2004). In particular, RTM protein powders represent a rapidly growing segment of the sports nutrition and dietary supplement industries, where whey protein ingredients dominate the market due to their high protein quality, rapid digestibility, and desirable functional properties (Kheto et al. 2025). RTM powders are widely used in sports nutrition and dietary supplements because they provide shelf‐stable, high‐protein beverages that consumers can easily reconstitute before consumption. Consequently, there is increasing interest in identifying alternative or complementary whey‐derived ingredients that can enhance functionality while supporting sustainable dairy processing.

WPPC is a coproduct generated during WPI manufacture through membrane fractionation of WPC (Burrington et al. 2014; Levin et al. 2016). According to the American Dairy Products Institute (ADPI) standard, WPPC typically contains ≥50% protein, ≥12% fat, ≤8% ash, and ≤6% moisture (dry basis) (ADPI 2023). WPPC contains a unique combination of whey proteins and milk fat globule membrane (MFGM)‐associated proteins and lipids, including phospholipids such as phosphatidylcholine (Ozturk et al. 2022), making it a unique dairy ingredient with both functional and nutritional potential. Compared with conventional WPI, which typically contains more than 90% protein and minimal fat, WPPC retains appreciable amounts of residual lipids and MFGM components, giving it a distinct compositional profile and functionality. Despite its valuable composition, WPPC remains underutilized, and expanding its food applications could improve coproduct utilization and support sustainability within dairy processing.

Previous studies have demonstrated that these components contribute to functional properties such as emulsification and water‐holding capacity, distinguishing WPPC from conventional whey protein ingredients (Levin et al. 2016; Swaminathan et al. 2021). Despite being enriched in nutritionally valuable components, WPPC remains underutilized and is used primarily in animal feed (ADPI 2023) with limited applications in foods, including as an emulsifying ingredient (Levin et al. 2016). Its performance in complete beverage formulations has not been comprehensively characterized.

Functional performance in beverage applications is strongly influenced by pH, protein‐lipid interactions, and processing conditions. Whey protein systems exhibit reduced stability near their isoelectric point, leading to aggregation, sedimentation, and diminished consumer acceptance (Levin et al. 2016). The presence of phospholipids and residual fat in WPPC may be associated with differences in functional behavior through effects on dispersion characteristics and interfacial interactions; however, the specific mechanisms were not evaluated in the present study.

Previous work has reported pH‐dependent differences in WPPC solubility, heat stability, and emulsifying behavior (Levin et al. 2016); however, direct comparisons with commercial WPI beverages that integrate both physicochemical measurements and sensory evaluation remain limited. Although WPC represents the largest share of the whey protein ingredient market due to its affordability, WPI is more commonly used in ready‐to‐mix (RTM) and ready‐to‐drink (RTD) protein beverages because of its higher protein purity, lower lactose content, and greater formulation stability (Mordor Intell. 2026). For this reason, a commercially available WPI‐based RTM beverage was selected as a commercially relevant comparator for the WPPC‐based RTM formulation in this study. Because the two formulations were not compositionally matched, comparisons between them were intended to characterize the performance of the complete beverage formulations rather than isolate effects attributable specifically to WPPC or WPI.

In addition to its functional attributes, WPPC contains nutritionally relevant phospholipid‐associated choline, which has generated interest in WPPC as a value‐added dairy ingredient (Ozturk et al. 2022; Zeisel, 2006). In our previous work, phospholipids accounted for approximately 20% of the total fatty acid pool in WPPC (Ozturk et al. 2022). Among these, phosphatidylcholine (PC) and phosphatidylethanolamine (PE) were the most abundant phospholipids (∼9 mg/g each based on fatty acid content), followed by lower concentrations of phosphatidylserine (PS), phosphatidylinositol (PI), and sphingomyelin (SM). Phosphatidylcholine is a major dietary source of choline. Choline is an essential nutrient involved in maintaining structural integrity and cell membrane signaling and a precursor for acetylcholine, a key neurotransmitter (Kenny et al., 2025). These findings highlight WPPC as a potential source of phospholipid‐associated choline for future food applications and provide additional context for its utilization. Despite established Adequate Intake (AI) recommendations of 425 mg/day choline for women and 550 mg/day for men, many adults do not meet these intake levels. Therefore, ingredients that naturally contain choline have attracted interest in food and beverage applications. However, information regarding the bioavailability of choline from WPPC‐based foods remains limited.

Despite the compositional uniqueness of WPPC and its potential functionality derived from MFGM components, limited information is available regarding its performance in RTM beverage systems that integrate ingredient functionality, rheological behavior, and consumer sensory perception. Most previous studies have focused on compositional characterization or individual functional attributes of WPPC rather than evaluating its application within a complete beverage formulation. Therefore, systematic assessment of WPPC in RTM beverage systems is needed to better understand its behavior during reconstitution and its suitability for use in protein beverages and nutritional supplement formulations. Characterizing the functional properties of WPPC in model aqueous systems is an important first step toward understanding its potential as a food ingredient. Accordingly, the functional measurements reported in this study should be interpreted as ingredient‐level characterization rather than direct predictors of behavior in milk‐based beverage systems. However, the behavior of WPPC in a complex matrix, such as milk containing caseins, fat globules, and minerals, may differ due to additional protein–protein and protein–lipid interactions. Therefore, both ingredient‐level characterization and application‐based evaluation are necessary for future comprehensive assessment.

Therefore, the objective of this study was to formulate a WPPC‐based RTM beverage powder and systematically evaluate its functional, physicochemical, rheological, and sensory properties in comparison with a commercially available WPI RTM beverage formulation. Specifically, the effects of pH on water‐holding capacity, heat stability, apparent emulsifying behavior, sedimentation, particle size, zeta potential, and microscopic characterization were examined. This work provides formulation‐relevant insights into the use of WPPC in protein beverage systems and supports the valorization of this underutilized dairy coproduct.

## 2 Materials and Methods

### 2.1 Materials

WPPC powder was kindly provided by Hilmar Ingredients (Ca 95324 USA). A commercial WPI powder (Isopure, Dutch Chocolate, low‐carbohydrate formulation) was used as a reference product for comparative functional, rheological, and sensory evaluations. Additional ingredients used in beverage formulation included fructose (BulkSupplements, USA), sea salt (Amazon Grocery), fat‐reduced cocoa extract and natural vanilla flavor (Prova, USA), cocoa powder (Olam Food Ingredients, USA), canola oil powder (CORE Ingredients, USA), and monohydrate dextrose (ADM, USA). The commercial RTM comparator was Isopure Low Carb Protein Powder (Dutch Chocolate; Glanbia Performance Nutrition, USA), containing whey protein isolate as the primary protein source together with cocoa powder, natural flavors, soy lecithin, xanthan gum, sucralose, vitamins, and minerals. Pasteurized 2% fat milk was purchased from a local supermarket. All dry ingredients were stored at ambient temperature until use. Sodium phosphate dibasic heptahydrate, sodium phosphate monobasic monohydrate, sodium acetate trihydrate, and glacial acetic acid were obtained from Sigma‐Aldrich (St. Louis, MO, USA) and used for buffer preparation and pH adjustment. A 40 g sample of each WPPC powder was labeled with a three‐digit code, and the samples were sent to an external lab (Eurofins, CA, 93722, USA) for the total choline analysis.

### 2.2 WPPC‐Based Beverage Formulation

The WPPC‐based beverage formulation was developed using WPPC powder obtained from Hilmar Ingredients (Hilmar, CA, USA). The composition of the WPPC powder was consistent with specifications established by the American Dairy Products Institute for human food applications (ADPI 2023). Formulation development was informed by compositional analysis of the WPPC powder, with particular emphasis on phospholipid‐associated choline content. The target choline content was selected to ensure a consistent and quantifiable choline contribution per serving. The WPPC‐based RTM formulation was designed to provide approximately 46 mg choline per prepared serving (100 g). This level was selected to contribute toward the estimated gap between typical dietary choline intake and the AI recommendation for women aged 51–70 years.

Beverage formulation was developed at the Center for Dairy Research (CDR), University of Wisconsin–Madison. The WPPC RTM powder consisted of WPPC (63.68 g), fructose (18.42 g), fat‐reduced cocoa powder (1.52 g), cocoa extract (2.47 g), natural vanilla flavor (1.52 g), sea salt (0.38 g), dextrose powder (7.21 g), and canola oil powder (4.79 g) (Table 1). All dry ingredients were weighed and blended thoroughly to produce a homogeneous RTM beverage mix. When 45.70 g of the developed WPPC RTM powder was reconstituted with 246 g of 2% milk, the resulting beverage contained 29.2 g total protein. For nutrition labeling purposes, the prepared beverage provided 10 g protein per 100 g serving. Similarly, when the commercial WPI RTM powder was reconstituted with 236.59 g of 2% milk, the resulting beverage contained 42 g total protein. For nutrition labeling purposes, the prepared beverage provided 14 g protein per 94 g serving (Table 2).

**TABLE 1:** WPPC RTM powder for the beverage system's formulation.

Ingredient	Amount (g)	Source
WPPC powder	63.68	Hilmar ingredients
Fructose	18.42	BulkSupplements
Dextrose powder	7.21	ADM
Canola oil powder	4.79	CORE ingredients
Cocoa extract	2.47	Prova
Fat‐reduced cocoa powder	1.52	Ofi
Natural vanilla flavor	1.52	Prova
Sea salt	0.38g	Amazon grocery

**TABLE 2:** Composition and nutritional characteristics of the WPPC‐based and commercial WPI‐based RTM beverages.

Parameter	WPPC‐based RTM beverage	Commercial WPI‐based RTM beverage
RTM powder used (g)	45.70	45.70
2% milk used for reconstitution (g)	246.00	236.59
Prepared beverage weight (g)	291.70	282.29
Nutrition Facts serving size (g)	100	94
Protein per serving (g)	10	14
Total protein in prepared beverage (g)	29.17	42
Protein contributed by 2% milk (g)	∼8.36	∼8.04
Protein contributed by RTM powder (g)	20.8	34.0
		
Total fat (g)	4	2
Total carbohydrate (g)	9	5
Total sugars (g)	8	4
Added sugars (g)	4	0
Sodium (mg)	65	35
Calories (kcal)	110	90
Choline per serving (mg)	46	ND

*Note*: Values were calculated from ingredient specifications and formulation amounts. Protein contributions from the RTM powder and 2% milk are presented separately to clarify total protein in the prepared beverage. “Protein per serving” refers to the Nutrition Facts serving size (100 g for the WPPC beverage and 94 g for the WPI beverage), whereas “total protein” refers to the entire prepared beverage.

Abbreviation: ND, not determined.

The RTM powder was produced as a single batch, packaged in airtight containers, and stored at room temperature until analysis. The commercial WPI RTM powder (Isopure, Dutch Chocolate, low‐carb) was selected as a benchmark because it represents a commercially available high‐protein RTM product commonly used in the sports nutrition market. During the formulation of the WPPC RTM powder, ingredient levels were adjusted to achieve a beverage system as comparable as possible to the commercial product in terms of composition and intended use. Functional, physicochemical, and rheological evaluations were conducted using subsamples obtained from this batch. For each analysis, three independently prepared dispersions were generated from the RTM powder and subsequently analyzed in triplicate. Therefore, the study included sample preparation and analytical replicates but did not include replication across independent production lots. For sensory evaluation, the RTM powders were reconstituted with pasteurized 2% milk immediately prior to testing. The RTM powder was combined with milk and mixed using a kitchen blender for 4–5 min until fully dispersed. The beverage was prepared on the same day as sensory testing to ensure consistency of product quality (Table 2).

### 2.3 Sample Preparation

WPPC RTM powder and commercial WPI RTM powder were used to prepare samples for functional, physicochemical, rheological, and sensory properties evaluations. Unless otherwise stated, samples were prepared fresh on the day of analysis.

Functional and rheological analyses were conducted using dispersions prepared from the RTM powders in water to evaluate the intrinsic functionality of the formulated powder systems independent of the milk matrix. For these analyses, RTM powders were hydrated in water at the concentrations specified for each test. Functional property measurements were conducted at three pH conditions: pH (4.5), pH (7.0), and native (unbuffered), defined as the intrinsic pH of the dispersion without pH adjustment. The native (unbuffered) pH of the dispersions ranged from 6.35 to 6.51. After hydration, pH adjustment was performed using appropriate buffer systems. Phosphate buffer was used to adjust samples to pH (7.0), and acetate buffer was used to adjust samples to pH (4.5). pH values were verified using a calibrated pH meter prior to analysis.

To ensure comparability between formulations, the protein concentration of the WPPC and WPI dispersions was standardized prior to analysis by adjusting the amount of RTM powder added to water according to the protein content of each powder so that equivalent protein concentrations were obtained. RTM powders originated from a single production lot. For each analysis, three independently prepared dispersions were evaluated, and measurements were conducted in triplicate. Results are reported as mean values ± standard deviation.

In contrast, sensory evaluation was conducted using beverages reconstituted with pasteurized 2% milk, as described in Section 2.2, to represent the intended consumer preparation of the RTM beverage system. This approach allowed instrumental measurements to characterize ingredient functionality under controlled dispersion conditions while sensory evaluation assessed consumer perception of the final prepared beverage.

## 3 Functionality Evaluation

WPPC is a nutritionally valuable ingredient with desirable functional properties for food applications. The functional behavior of whey protein ingredients, including WPI and WPC, is strongly influenced by pH due to changes in protein charge, solubility, and aggregation behavior (Lam and Nickerson 2015; Ghanimah and Ibrahim 2018; Chen et al. 2019). According to Liu et al. (2022), pH modulates WPC gel properties by changing the charge on protein molecules, thereby affecting gel structure and interactions (Liu et al. 2022). The approach of using multiple pH rather than a single pH level in this study enables us to have a more comprehensive understanding of protein‐protein and protein‐lipid interactions in WPPC‐based systems and allows identification of conditions that optimize functional performance. Furthermore, evaluating functionality across different pH levels reflects the variability encountered in real food systems and practical beverage formulations. Therefore, functional properties were evaluated across different pH conditions, including (4.5), (7.0), and native (unbuffered), to provide broader characterization of WPPC as an ingredient for a wide range of beverages, including water, milk, plant‐based beverages, smoothies, and fruit‐based beverages and to facilitate comparison with previous studies of whey‐derived ingredients. Key functional properties evaluated in this study included water‐holding capacity (WHC), heat stability index (HSI), emulsifying activity and stability, apparent solubility, and sedimentation, which collectively describe the behavior of WPPC in beverage systems. Functional measurements were performed on dispersions prepared from RTM powders in water to isolate ingredient functionality independent of the milk matrix used for sensory evaluation.

### 3.1 Water‐Holding Capacity

Water‐holding capacity (WHC) of the WPPC and WPI RTM powders was determined according to the method described by Levin et al. (2016). Briefly, the powders were hydrated at 10% (w/v), and pH was adjusted using the relevant buffers to pH 4.5 and 7.0 or native (unbuffered). The solutions were transferred into pre‐weighted 15‐mL capped plastic tubes, stirred using a vortex, and incubated at room temperature for 1 h. The samples were centrifuged at 2,000 × *g* for 30 min. The supernatant was decanted into labeled flasks and placed in a 100°C oven for 24 h to remove all water. The pellet from the supernatant was weighed. Residual buffer or water in the tube and dry solids from the buffer were calculated by performing the process without the powders. The WHC (%) was determined as the percentage of the initial water remaining in the gel and calculated using  Equation [1] (Neumann et al. 1984). All measurements were conducted in triplicate.

$$\begin{array}{ccc} \mathrm{WHC} & = & \left[\mathrm{wet}\;\mathrm{pelletwt}\left(g\right)-\mathrm{buffer}\;\mathrm{retained}\;\mathrm{by}\;\mathrm{blankwt}\left(g\right)\right]\\ & & /\left[\mathrm{total}\;\mathrm{samplewt}\left(g\right)-\mathrm{decanted}\;\mathrm{solidswt}\left(g\right)\right.\\ & & \left.-\;\mathrm{solids}\;\mathrm{from}\;\mathrm{bufferwt}\left(g\right)\right] \end{array}$$

### 3.2 Heat Stability Index

Heat stability index (HSI) of the WPPC and WPI RTM powders were evaluated following the method of Levin et al. (2016). pH of the samples was adjusted for (4.5), (7.0), or native (unbuffered). A volume of 10 mL of each solution was transferred to the labeled 15‐mL pre‐weighed centrifuge tubes, and the weight of samples were recorded. The samples were heated using water bath at 85°C for 3 min, and the tubes were immediately cooled in an ice bath for 10 min. Following cooling, the samples were centrifuged at 2800 g for 15 min. The pellet was weighed, and the HSI was calculated according to Equation [2] (Harper and Lee 1998). All measurements were conducted in triplicate. HSI was calculated as the percentage of precipitated material after heating and centrifugation; therefore, higher HSI values indicate greater protein precipitation and lower heat stability.

$$\mathrm{HSI}\;\left(\%\right)=\frac{\mathrm{pelletwt}\left(g\right)}{10\;\mathrm{ml}\;\mathrm{solutionwt}\left(g\right)}\;\;\times 100$$

For ease of interpretation, heat stability results are presented in the Results and Discussion as the percentage of powder remaining in solution, calculated as:

Powder remaining in solution (%) = 100−HSI (%)

Accordingly, higher values for powder remaining in solution indicate lower precipitation and greater heat stability.

### 3.3 Emulsifying Activity Index and Emulsifying Stability Index

Emulsifying Activity Index (EAI) and Emulsifying Stability Index (ESI) of the WPPC and WPI RTM powders were determined using a turbidimetric method as described by (Fan et al. 2021). Protein solutions were prepared at a concentration of 2.0 g/L and adjusted to pH (4.5), (7.0), or native (unbuffered). Emulsions were prepared by homogenizing canola oil and aqueous phase solutions at a ratio of 20:80 (w/w) using a high‐speed homogenizer (OMNI Mixer) at 36200 × g for 2 min. Immediately after homogenization, aliquots of the emulsion were diluted 50‐fold with 1.0 g/L sodium dodecyl sulfate (SDS) solution and vortexed. Absorbance was measured at 500 nm using a UV‐vis spectrophotometer (Thermo Scientific Genesys 20). A second aliquot was stored at room temperature for 10 min prior to dilution and absorbance measurement. EAI and ESI were calculated using Equations (3) and (4), respectively. All measurements were performed in triplicate.

(3) $$\mathrm{EAI}\left(m^{2}/g\right)=\mathrm{DF}\times \left(2\;\times 2.303\;\times A0\right)/\left(c\times \left(1-\phi \right)\times 10^{4\;}\right)$$

(4) $$\mathrm{ESI}\;\left(\min\right)=\;A0/\left(A0-A10\right)\times t$$

where, 2: a constant because the interfacial area of an emulsion is twice its turbidity

2.303: the value ln (10)

DF: the dilution factor (50)

c: the present protein concentration in the aqueous phase (g/mL)

Φ: the oil volume fraction (0.2)

t: the time interval (10 min)

A0 and A10: the absorbance of diluted emulsion at 0 and 10 min, respectively.

### 3.4 Apparent Solubility

The apparent solubility index was determined according to the method described by (Ismail et al. 2020) with slight modification. Protein dispersions (5% w/v) of the WPPC RTM and WPI powders were adjusted to pH (4.5), (7.0), or native (unbuffered) and mixed at room temperature for 30 min. Samples were then centrifuged at 3,680 × g for 10 min. The volume of sediment formed at the bottom of the centrifuge tube was recorded. Solubility was calculated using the following Equation (5). All measurements were performed in triplicate. This measurement reflects apparent dispersion behavior based on sediment volume after centrifugation, rather than protein solubility.

$$\mathrm{Solubility}\;\left(\%\right)=\frac{\mathrm{supernatant}\;\mathrm{volume}}{\mathrm{Total}\;\mathrm{volume}}\times 100$$

### 3.5 Sedimentation

Sedimentation behavior of the WPPC and WPI RTM powders was evaluated using a method described by (Ismail et al. 2020). The dispersions (5% w/v) were prepared and adjusted to pH (4.5), (7.0), or native (unbuffered). Following mixing and centrifugation under the same conditions used for solubility testing, sediment volume was recorded and used as an indicator of sedimentation propensity. All measurements were performed in triplicate.

## 4 Analysis of Particle Size and Zeta‐Potential

Particle size and zeta‐Potential of WPPC and WPI RTM dispersions were evaluated by dynamic light scattering (DLS) using a 90 Plus particle size analyzer (Brookhaven Instruments Corporation, New York 11742, USA). The WPPC and WPI RTM dispersions (5% w/v) were prepared and adjusted to pH (4.5), (7.0), or native (unbuffered) and stirred continuously at room temperature (Swaminathan et al. 2021). All measurements were done in triplicate.

## 5 Microscopic Evaluation

All microscope images of WPPC and WPI RTM samples were viewed using a Nikon Labphot‐2 light microscope (Melville, NY) at 40× magnification. Five random images were taken per sample. WPPC and WPI dispersions (10%, w/v) were prepared in deionized water and stained with 0.1% (w/v) eosin Y prior to microscopic evaluation (Fisher Scientific, Waltham, MA) (Levin et al. 2016).

## 6 Rheological Evaluation

### 6.1 Apparent Viscosity

The rheological behavior of the reconstituted WPPC and the commercial WPI RTM powders was evaluated using a rotational Brookfield viscometer (Brookfield Engineering Laboratories, Middleboro, MA, USA) equipped with an SC4–21 spindle (Benoit et al., 2013). Protein dispersions were prepared at 10% (w/w) protein concentration and adjusted to pH (7.0) using phosphate buffer. Samples were stirred at 112×*g* for 1 h at room temperature to ensure complete hydration and then stored at 4°C overnight. Prior to measurement, samples were equilibrated to room temperature (22 ± 1°C) and stirred for an additional 1 h. Approximately 16 mL of each sample was transferred to the viscometer sample chamber. Apparent viscosity measurements were conducted at a spindle speed of 10 rpm, and viscosity values were recorded after 30 s of shear. All measurements were performed in triplicate using independently prepared samples. Flow behavior data were fitted to the power‐law model:

$$\eta a=K\gamma^{n-1}$$

where ηa is apparent viscosity (mPa.s), *γ* is shear rate (s‐1), *K* is consistency coefficient (Pa.sn), and *n* is the flow behavior index (dimensionless). Values of *K* and *n* were determined by nonlinear regression.

## 7 Color Analysis (or Colorimetry)

Color measurements of the reconstituted WPPC and the WPI RTM powders were performed using a HunterLab ColorFlex EZ colorimeter (Hunter Associates Laboratory, Reston, VA, USA). Protein solutions were prepared at 5% (w/v) and evaluated at native (unbuffered) pH. Color values were recorded using the CIE Lab* color space, where L* represents lightness (0 = black, 100 = white), a* represents the red–green axis, and b* represents the yellow–blue axis. For each sample, three independent measurements were collected, and mean values were reported. Measurements were conducted under standardized ambient lighting conditions.

## 8 Sensory Evaluation

Consumer sensory evaluation of the reconstituted WPPC and WPI RTM beverages was conducted in accordance with an exempt protocol approved by the Institutional Review Board (IRB) of the University of Wisconsin–Madison (IRB Protocol no. 2025‐0245). A total of 100 untrained consumers were recruited from faculty, staff, and students in the Department of Food Science in Madison, WI, as well as members of the general public. Sensory evaluations were conducted over three consecutive days. Before the sensory session, participants reviewed and signed a consent information sheet outlining the objectives and procedures of the study. Participants were informed that they would evaluate and compare two chocolate‐flavored whey protein‐based beverages containing milk and were asked to disclose any relevant allergies before participation. The beverages were prepared within 30 min prior to each evaluation session as described previously (Section 2.2), stored at 4°C, and equilibrated to serving temperature (22 ± 1°C) approximately 5 min before presentation. Samples (20 mL) were served in polystyrene cups labeled with randomized three‐digit codes and presented in individual sensory booths illuminated with yellow lighting. Both beverage samples were presented to each panelist during the session. To minimize order effects, the presentation order of the samples was randomized and counterbalanced across panelists, such that approximately half of the participants received the WPPC beverage first and the other half received the WPI beverage first. Panelists were instructed to taste both samples and cleanse their palate with water between samples. Consumer liking of appearance, texture, flavor, aftertaste, overall liking, color, and aroma was evaluated using a 9‐point hedonic scale, where 1 = dislike extremely and 9 = like extremely (Drake 2007). Purchase intent was assessed separately at the end of the questionnaire using a 5‐point purchase‐intent scale with the following response options: (1) Definitely would not buy it, (2) Probably would not buy it, (3) might or might not but, (4) Probably would buy it, (5) Definitely would buy it.

Participants recorded their responses using paper questionnaires administered during the sensory evaluation sessions. Completed questionnaires were collected immediately following evaluation and subsequently entered into an electronic database for statistical analysis. Demographic information, including age and gender, was collected prior to testing.

## 9 Statistical Analysis

All measurements were performed in triplicate, and results are reported as mean ± standard deviation. One‐way analysis of variance (ANOVA), followed by Tukey's post hoc test, was performed separately to evaluate the effect of pH within each system at a 95% confidence level using GraphPad Prism version 10 (GraphPad Software Inc., La Jolla, CA, USA) and R software (version 4.6.0). This statistical approach was selected because the primary objective of the study was to evaluate pH‐dependent changes within each RTM formulation rather than to assess formulation × pH interaction effects. Consumer liking scores for appearance, color, aroma, texture, flavor, aftertaste, and overall liking were analyzed using linear mixed‐effects models, with beverage formulation included as a fixed effect and participant included as a random effect to account for the paired nature of the sensory evaluation, in which each participant evaluated both beverage formulations. Purchase‐intent scores were analyzed separately using the same linear mixed‐effects modeling approach. Statistical significance was defined as *p* < 0.05. The model was specified as:

$$\;Score_{ij}=\mu +\beta .\mathrm{Produc}t_{i}+u_{j}+e_{ij}$$

where μ is the overall mean, $\beta .\mathrm{Produc}t_{i}$ is the fixed effect of product formulation, $u_{j}∼N\left(0,\sigma_{panelist}^{2}\right)$ is the random effect representing participant‐specific variation and $e_{ij}∼N\left(0,\sigma^{2}\right)$ is the residual error.

## 10 Results and Discussion

### 10.1 Water‐Holding Capacity

Water‐holding capacity (WHC) is an important functional property for protein‐based beverage powders, as it influences hydration behavior, physical stability, and mouthfeel during reconstitution (Kneifel and Seiler 1993; Levin et al. 2016). The WHC of the WPPC and the WPI RTM powders were evaluated at pH (4.5), (7.0), and native (unbuffered) pH, and the results are shown in Figure 1. WHC was expressed as a percentage (%) of water retained.

**FIGURE 1:**
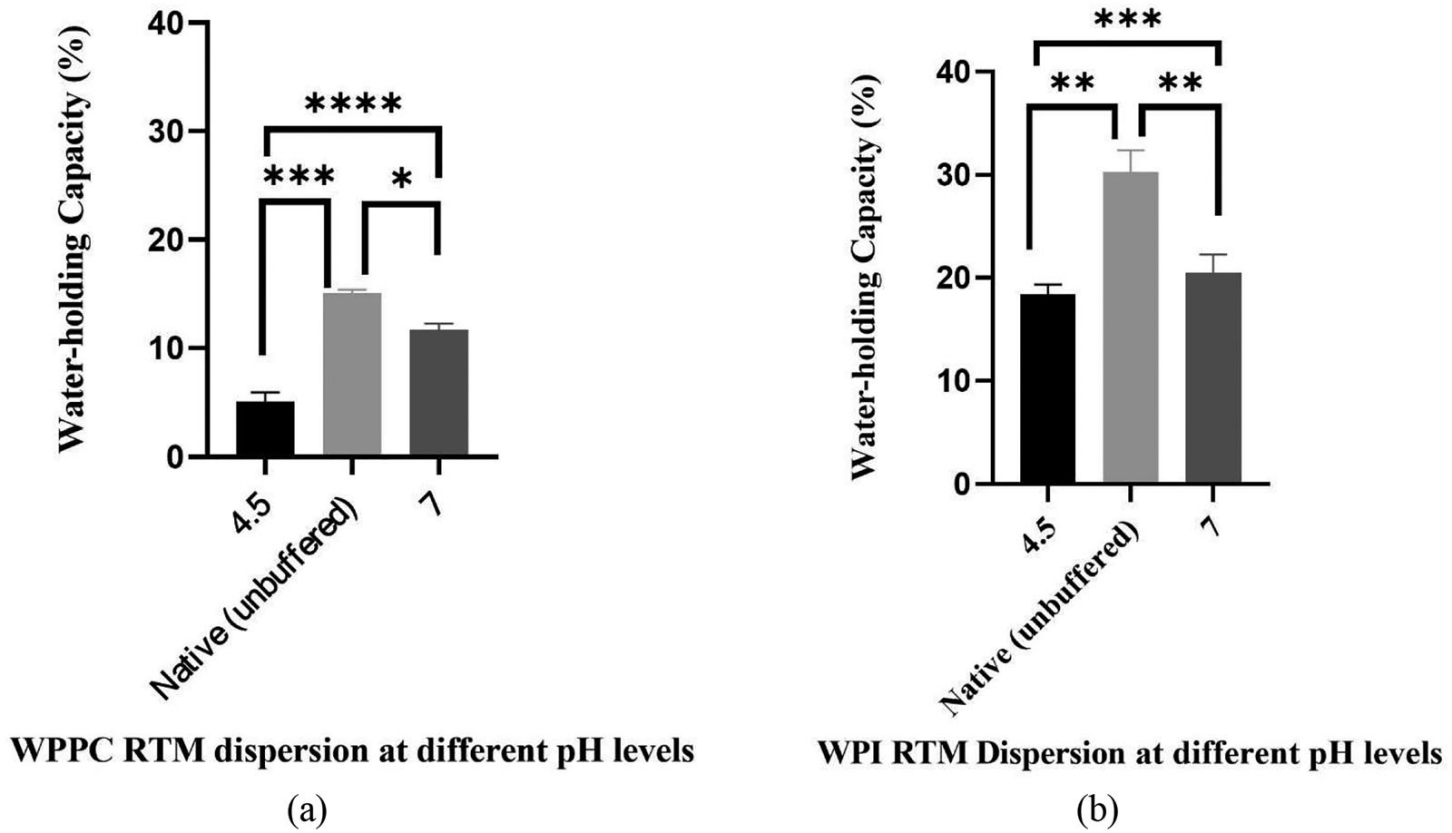
Water‐holding Capacity (grams of water retained in the sediment) across (a) the WPPC RTM dispersion and (b) the WPI RTM dispersion at three different pH levels (4.5), (7.0), and native (unbuffered). Error bars represent Error bars represent the Standard Error of the Mean (SEM). Statistical differences were determined using one‐way analysis of variance (ANOVA) followed by Tukey's post hoc test. Asterisks indicate significant differences (*p < 0.05, **p < 0.01, ***< 0.001, **** < 0.0001), and the comparisons are across pH within each formulation.

For both WPPC and WPI RTM powders, WHC was significantly affected by pH (*p* < 0.05). The lowest WHC values were observed at pH (4.5), while higher WHC was measured at pH (7.0) and native (unbuffered). This trend is consistent with the behavior of whey protein systems near their isoelectric point, where reduced net charge promotes protein aggregation and limits the ability of the protein matrix to retain water. Similar pH‐dependent reductions in WHC for whey protein ingredients have been reported previously (Levin et al. 2016).

Notably, both RTM powders exhibited higher WHC at native (unbuffered) compared with pH (7.0). However, at pH native (unbuffered) and (7.0), the WPI RTM powder exhibited higher WHC compared to the WPPC RTM powder. This finding is likely attributable to the higher protein purity and lower lipid content of the WPI RTM powder, which may promote the formation of a more continuous protein network capable of entrapping water.

In contrast, the presence of phospholipids and residual fat in the WPPC RTM powder may have contributed to partially disrupting protein–protein interactions, resulting in a less compact gel structure and reduced water retention. This behavior has been reported previously for WPPC systems and may reflect differences in ionic strength or mineral interactions associated with buffer composition at neutral pH, which can influence protein hydration and gel structure (Chantrapornchai and McClements 2002; Levin et al. 2016). Despite this difference, the WHC observed for the WPPC RTM powder remained within a functional range suitable for beverage applications (Heiden‐Hecht et al. 2026; Nik et al. 2010). These results highlight the sensitivity of WHC to pH and formulation conditions and underscore the importance of pH control when incorporating WPPC into beverage systems, particularly for acidified beverages such as fruit‐flavored protein waters, sports recovery drinks, juice‐protein blends, and nutritional beverages, where protein aggregation and instability at pH 4.5 may negatively affect product appearance, mouthfeel, and sedimentation behavior. Understanding these pH‐dependent changes can assist manufacturers in optimizing formulation strategies for WPPC‐containing beverages.

It should be noted that samples adjusted to pH 4.5 and pH 7.0 were prepared using acetate and phosphate buffers, respectively, whereas native samples were unbuffered. Therefore, the observed differences may reflect not only the effects of pH but also differences in buffer composition and ionic environment. Previous studies have demonstrated that salts, including phosphate‐based systems, can influence whey protein denaturation, hydration, and intermolecular interactions independently of pH by modifying ionic conditions and buffering capacity (Perez et al. 2022). Accordingly, the responses observed in the present study should be interpreted as the combined effects of pH and the associated buffer systems rather than pH alone.

Overall, while the WPPC RTM powder demonstrated lower WHC than the WPI RTM powder, its pH‐dependent hydration behavior was consistent with whey‐based protein systems and did not preclude its use in reconstituted beverage formulations. When considered alongside other functional attributes, including emulsifying properties and sensory performance, the WHC results support the feasibility of WPPC as a functional ingredient in protein beverages.

### 10.2 Heat Stability Index

Heat stability index (HSI) is a critical functional attribute for protein‐based beverage systems, as thermal processing can induce protein denaturation, aggregation, and precipitation, ultimately affecting product stability and consumer acceptability. HSI of WPPC RTM powder was evaluated because thermal tolerance reflects dispersion stability and structural integrity and may influence product performance under different preparation conditions, including incorporation into hot beverages (milk or water). For clarity, the results are presented as the percentage of powder remaining in dispersion (100−HSI), where higher values indicate less precipitation and greater heat stability. In addition, heat stability is a commonly reported functional parameter for dairy protein ingredients and facilitates comparison with existing literature. The HSI of the WPPC and the WPI RTM powders were evaluated at pH (4.5), (7.0), and Native (unbuffered), and the results are presented in Figure 2.

**FIGURE 2:**
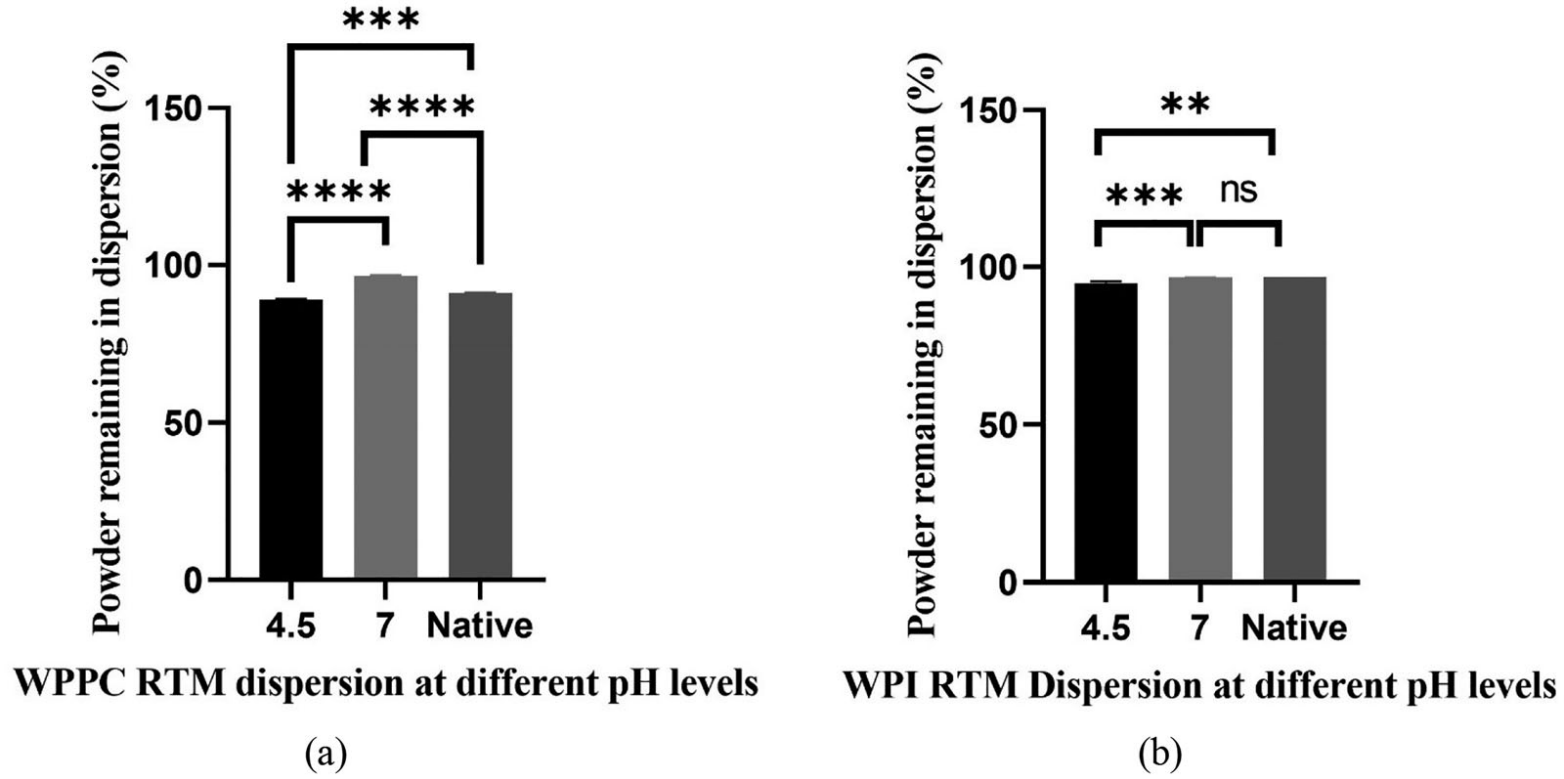
Percentage of powder remaining in dispersion after heat treatment for (a) the WPPC RTM dispersion and (b) the WPI RTM dispersion at three different pH levels (4.5), (7.0), and native (unbuffered). Higher values indicate lower precipitation and greater heat stability. Error bars represent the Standard Error of the Mean (SEM). Statistical differences were determined using one‐way analysis of variance (ANOVA) followed by Tukey's post hoc test. Asterisks indicate significant differences (*p < 0.05, **p < 0.01, ***< 0.001, **** <0.0001), and the comparisons are across pH within each formulation.

For the WPPC RTM powder, heat stability was significantly influenced by pH (*p* < 0.05). The lowest heat stability was observed at pH (4.5). Reduced heat stability at pH (4.5) is consistent with the proximity of whey proteins to their isoelectric point, where decreased electrostatic repulsion promotes protein–protein interactions and aggregation upon heating. Similar pH‐dependent reductions in heat stability of WPPC systems have been reported previously (Levin et al. 2016). The percentage of powder remaining in dispersion increased from pH 4.5 to native (unbuffered) pH and reached its highest value at pH 7.0, indicating greater heat stability under neutral conditions. At neutral and native (unbuffered) pH, the WPPC RTM powder exhibited high heat stability, indicating that the system remained largely resistant to thermal precipitation under these conditions, and the greater stability observed at neutral pH may be attributed to increased electrostatic repulsion between proteins, which supports dispersion during heating.

The WPI RTM powder exhibited a high percentage of powder remaining in dispersion at pH 7.0 and native (unbuffered) pH at pH (7.0) and native (unbuffered) pH, with significantly reduced stability only at pH 4.5. The relatively small difference in the percentage of powder remaining in dispersion between pH 7.0 and native (unbuffered) pH for the WPI RTM powder is consistent with previous reports indicating that WPI exhibits minimal variation in thermal stability across near‐neutral pH values (Buggy et al. 2018). This behavior reflects the dominance of intermolecular interactions and protein concentration effects over minor pH differences in this range.

Comparatively, the WPI RTM powder exhibited a non‐significantly higher percentage of powder remaining in dispersion than the WPPC RTM powder at corresponding pH conditions. Nevertheless, WPPC RTM powder maintained high heat stability at neutral and native (unbuffered) pH.

Overall, the results highlight the strong pH dependence of thermal stability in both WPPC and WPI systems and emphasize the importance of formulation and pH selection when designing WPPC‐containing beverages. The high percentage of powder remaining in dispersion under neutral and native (unbuffered) conditions indicates favorable heat stability of the WPPC RTM powder under the conditions evaluated.

### 10.3 Emulsifying Activity Index and Emulsifying Stability Index

The Emulsifying Activity Index (EAI) and Emulsifying Stability Index (ESI) provide insight into the ability of protein‐based systems to adsorb at the oil–water interface and stabilize emulsions over time, which is particularly relevant for beverage formulations containing lipid components. The EAI and ESI of the WPPC and the WPI RTM powders were evaluated at pH 4.5, 7.0, and native (unbuffered), and the results are presented in Figure 3.

**FIGURE 3:**
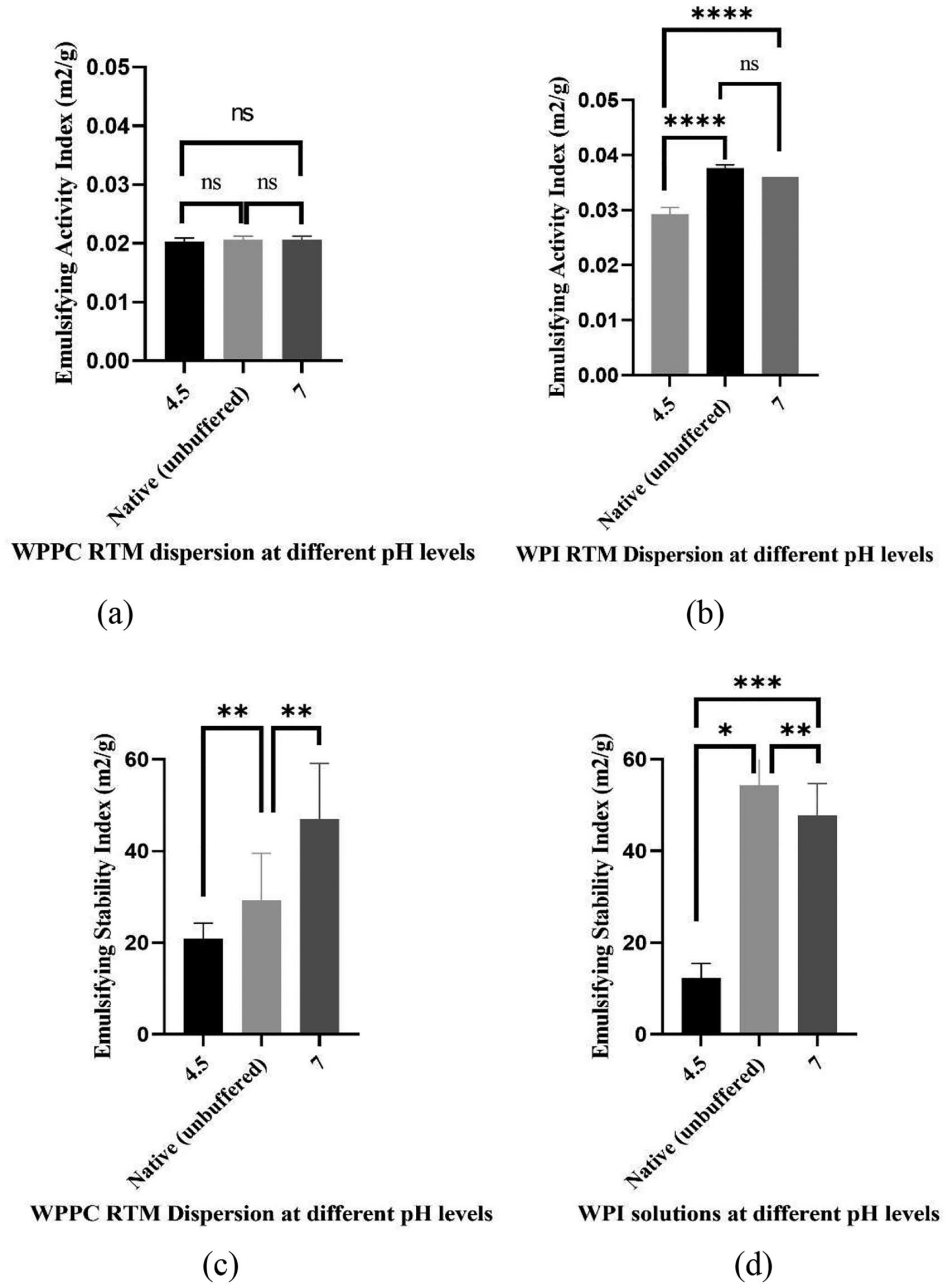
Emulsifying Activity Index of (a) the WPPC RTM dispersion and (b) the WPI RTM dispersion, and Emulsifying Stability Index of (c) the WPPC RTM dispersion and (d) the WPI RTM dispersion, at three different pH levels (4.5), (7.0), and native (unbuffered). Error bars represent the Standard Error of the Mean (SEM). Statistical differences were determined using one‐way analysis of variance (ANOVA) followed by Tukey's post hoc test. Asterisks indicate significant differences (*p < 0.05, **p < 0.01, ***< 0.001, **** <0.0001), and the comparisons are across pH within each formulation.

For the WPPC RTM powder, EAI did not differ significantly across the three pH conditions evaluated. This lack of a significant pH effect indicates that the measured EAI values of the WPPC system remained relatively stable over the pH range tested. In contrast, the WPI RTM powder exhibited a clear pH dependence in EAI, with significantly lower values at pH (4.5) compared with pH (7.0) and native (unbuffered). This trend is consistent with the reduced solubility and increased aggregation of whey proteins near their isoelectric point, which can limit protein availability for interfacial adsorption (Fan et al. 2021).

The ESI results further highlighted differences between the two systems. For the WPPC RTM powder, ESI was significantly higher at pH (7.0) than at pH (4.5) and native (unbuffered) pH, indicating improved emulsion stability under neutral conditions. The higher measured ESI at pH 7.0 is consistent with the greater electrostatic repulsion generally observed in whey protein systems at pH values farther from the isoelectric region, which can reduce droplet aggregation. The lower ESI observed at pH (4.5) is consistent with reduced net charge and increased protein–protein interactions, which can promote emulsion instability. The WPI RTM powder exhibited higher ESI values at native (unbuffered) and pH (7.0) compared with acidic conditions, reflecting the strong influence of protein charge on emulsion stability. When far from the isoelectric point, whey proteins carry substantial net charge, increasing electrostatic repulsion between droplets and limiting aggregation. These observations align with previous studies demonstrating enhanced emulsion stability of WPI systems at pH values distant from the isoelectric point (Lam and Nickerson 2015).

Numerically, the WPI system generally exhibited higher ESI values than the WPPC system at corresponding pH levels. However, direct statistical comparisons between formulations were not performed because the statistical analyses were designed to evaluate pH‐dependent differences within each formulation. In addition, because the RTM formulations contained cocoa and other ingredients that may contribute to light scattering during absorbance measurements and formulation‐specific blanks were not included, the EAI and ESI results should be interpreted with caution. Therefore, the observed values are best interpreted as apparent emulsifying behavior of the complete RTM formulations rather than definitive measures attributable specifically to the protein ingredients.

Overall, the EAI and ESI results suggest differences in apparent emulsifying behavior between the WPPC‐ and WPI‐based formulations across the evaluated pH conditions. However, because formulation‐specific blanks were not included, these measurements should be interpreted with caution. Future studies using formulation‐specific blanks or simplified model systems are needed to better characterize the emulsifying functionality of WPPC.

### 10.4 Apparent Solubility

Apparent solubility is a key ingredient‐quality attribute and one of the critical functional attributes for protein‐based beverage systems, as it influences dispersibility, physical stability, and consumer perception following reconstitution. The pH of the WPPC and the WPI RTM powders war adjusted to 4.5, 7.0, and native (unbuffered), and the results are shown in Figure 4. These measurements were conducted on dispersions prepared from the RTM powders, rather than the final beverage prepared with milk.

**FIGURE 4:**
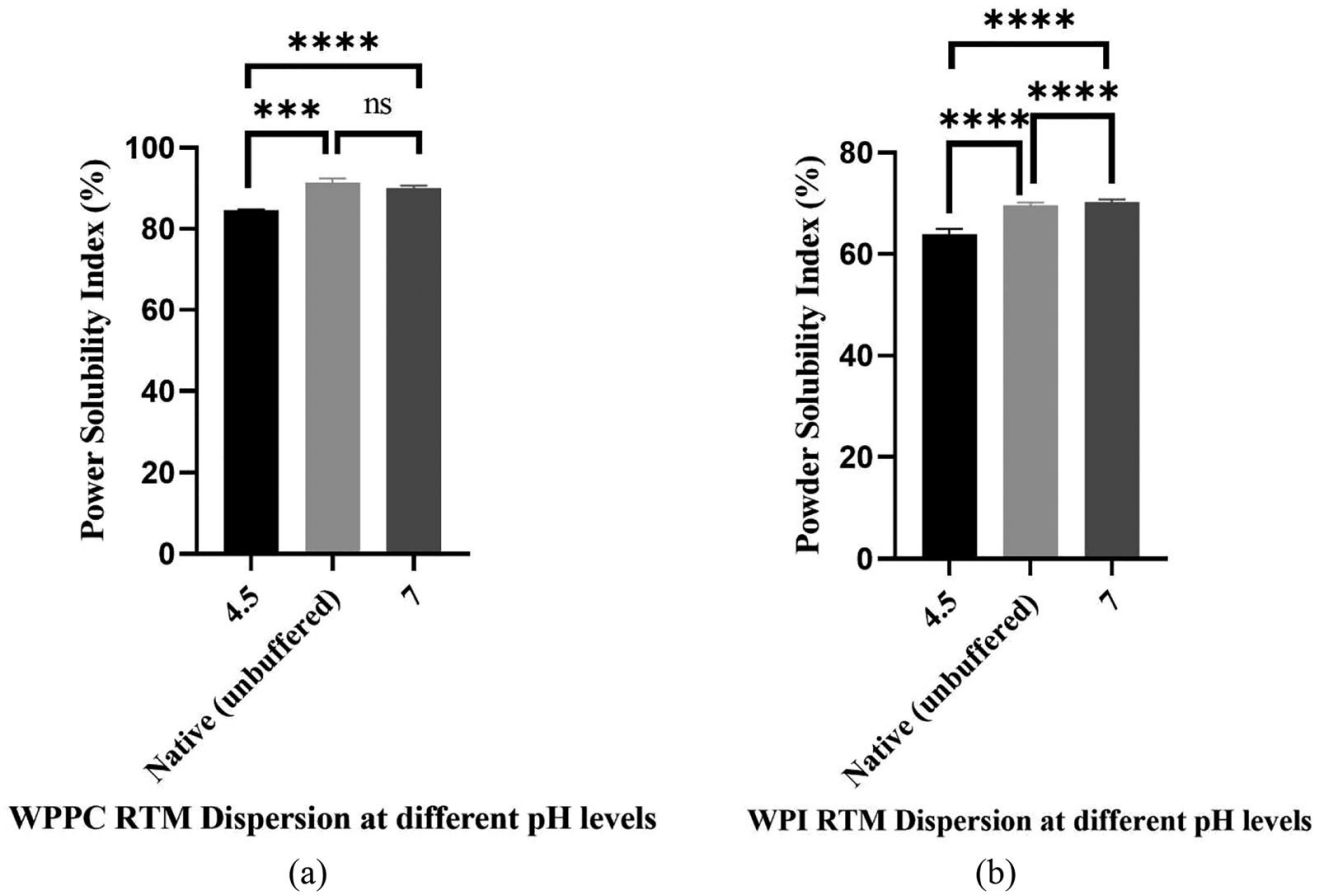
Solubility index of (a) WPPC RTM dispersion and (b) the WPI RTM dispersion at three different pH levels (4.5), (7.0), and native (unbuffered). Error bars represent the Standard Error of the Mean (SEM). Statistical differences were determined using one‐way analysis of variance (ANOVA) followed by Tukey's post hoc test. Asterisks indicate significant differences (*p < 0.05, **p < 0.01, ***< 0.001, **** < 0.0001), and the comparisons are across pH within each formulation.

For the WPPC RTM powder (Figure 4a), apparent solubility was significantly affected by pH (*p* < 0.05). The lowest solubility was observed at pH (4.5), while significantly higher values were found at pH (7.0) and native (unbuffered). This behavior is consistent with the proximity of whey proteins to their isoelectric point at pH (4.5), where reduced net charge promotes protein aggregation and limits protein–water interactions. As pH increased toward neutral and native (unbuffered) conditions, improved electrostatic repulsion and hydration resulted in enhanced solubility of the WPPC system.

The WPI RTM powder (Figure 4b) exhibited a similar pH‐dependent trend, with significantly reduced powder solubility at pH (4.5) and a higher value at pH (7.0) and native (unbuffered). However, at each pH condition, the solubility of the WPI RTM powder was consistently higher than that of the WPPC RTM powder. This difference may be partially attributed to differences in ingredient composition between the two RTM formulations. WPPC contains residual fat and phospholipids associated with MFGM fragments, whereas the WPI system contained lower lipid content. The presence of lipids and membrane material in WPPC may influence protein–water interactions and increase the likelihood of aggregation or sedimentation, resulting in lower measured solubility. In addition, the total fat content and other formulation components present in the RTM powders may also contribute to differences in dispersibility behavior.

These conditions are representative of many protein beverage formulations and demonstrate that the WPPC RTM powder achieved adequate dispersion under native and neutral pH conditions. However, the specific contribution of phospholipids to this behavior was not directly evaluated in the present study. Similar pH‐dependent solubility behavior has been reported for whey‐derived ingredients in previous studies (Levin et al. 2016).

Overall, the powder solubility profiles shown in Figure 4 demonstrate that while the WPI RTM exhibited superior solubility across all pH conditions, the WPPC RTM powder achieved adequate solubility at neutral and native (unbuffered) pH levels relevant to beverage applications. When considered alongside emulsifying performance and heat stability, these results support the feasibility of incorporating WPPC into reconstituted protein beverage systems.

### 10.5 Sedimentation

Sedimentation is a key consumer‐quality attribute that shows beverage stability during storage. Sedimentation behavior is an indicator of physical stability in reconstituted protein beverages, as excessive sediment formation can negatively affect appearance, mouthfeel, and overall consumer acceptance. The sedimentation of dispersions prepared from the WPPC RTM powder and the WPI RTM powder was evaluated at pH (4.5), pH (7.0), and native (unbuffered), and the results are presented in Figure 5.

**FIGURE 5:**
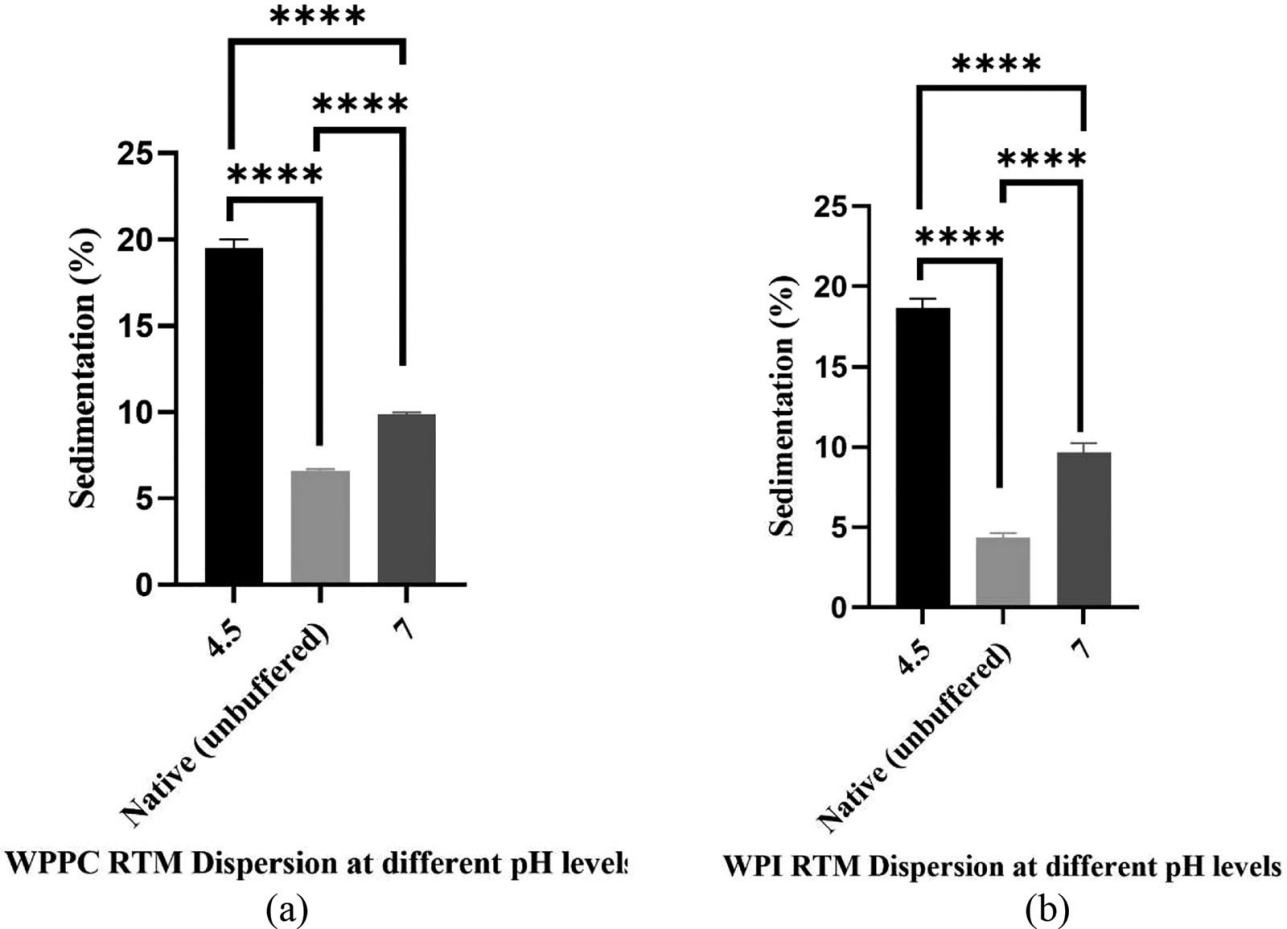
Sedimentation index of (a) WPPC RTM dispersion and (b) the WPI RTM dispersion at three different pH levels (4.5), (7.0), and native (unbuffered). Error bars represent the Standard Error of the Mean (SEM). Statistical differences were determined using one‐way analysis of variance (ANOVA) followed by Tukey's post hoc test. Asterisks indicate significant differences (*p < 0.05, **p < 0.01, ***< 0.001, **** <0.0001), and the comparisons are across pH within each formulation.

For both WPPC and WPI systems, sedimentation was strongly influenced by pH (*p* < 0.05). The highest sedimentation was observed at pH 4.5, while significantly lower sedimentation occurred at pH 7.0 and native (unbuffered). Increased sedimentation under acidic conditions is consistent with reduced electrostatic repulsion and increased protein aggregation near the isoelectric point of whey proteins, leading to the formation of larger, denser aggregates that settle more readily. Similar pH‐dependent sedimentation behavior has been reported for whey protein–based beverages (Ismail et al. 2020).

Across the tested pH conditions, sedimentation was highest at pH 4.5 for both beverage systems (*p* < 0.05), whereas significantly lower sedimentation was observed under native (unbuffered) and pH 7.0 conditions (Figure 5). At pH 4.5, the WPPC RTM dispersion exhibited slightly higher sedimentation (20.0%) than the WPI RTM dispersion (18.3%). However, because the two RTM beverage systems were not compositionally identical, this difference should be interpreted at the formulation level rather than attributed solely to the WPPC ingredient. WPPC contains residual lipids and MFGM‐derived components that may contribute to differences in aggregation behavior and dispersion stability. However, these mechanisms were not directly investigated in the present study.

It should be noted that the comparison in this study reflects differences between two formulated RTM beverage systems rather than purified protein ingredients alone, and therefore components such as lipids and other formulation ingredients present in the RTM powders may also influence sedimentation behavior. Despite exhibiting greater sedimentation than the WPI system, the WPPC system demonstrated substantially reduced sedimentation at pH (7.0) and native (unbuffered) pH compared with pH (4.5), indicating that sedimentation of WPPC beverages can be effectively minimized under near‐neutral pH conditions relevant to many protein beverage formulations. When considered together with the emulsifying and rheological results, the sedimentation behavior supports the feasibility of incorporating WPPC into RTM beverage formulations under near‐neutral pH conditions.

Overall, the sedimentation results highlight the critical role of pH in determining physical stability for both WPPC and WPI beverage systems and reinforce the importance of formulation design to optimize stability.

## 11 Analysis of Particle Size and Zeta Potential

### 11.1 Particle Size

Particle size influences the physical stability, mouthfeel, and reconstitution properties of protein‐based beverage powders. WPPC particle size affects creaminess and mouthfeel in dairy applications (Filla et al. 2021). One‐way ANOVA revealed that across all pH conditions, particle size was significantly affected by pH for both the WPPC and WPI RTM dispersions (*p* < 0.03 and *p* < 0.01, respectively). The WPPC and WPI RTM dispersions presented much larger particles near the isoelectric region at pH (4.5), demonstrating aggregation in both systems, although the WPI RTM dispersion exhibited larger mean particle sizes, approximately 1.7 times those in the WPPC RTM dispersion. (Wang et al. 2022). The size of aggregated WPPC was found to range from 20 to 150 µm by previous research (Swaminathan et al. 2021). In this study, particles larger than 100 µm were observed at pH 4.5 in both systems by microscopy. Smaller mean particle sizes were observed for both systems under native (unbuffered) and pH 7 conditions, indicating improved dispersion stability and reduced aggregation (Table 4). Although WPPC exhibited smaller particle sizes, WPI showed greater water‐holding capacity. This indicates that WHC depends not only on particle size but also on protein composition and the ability of proteins to bind wate**r**.

### 11.2 Zeta Potential (ZP)

Zeta potential (ZP) was strongly influenced by pH, with both WPPC and WPI RTM dispersions presenting lower values at pH 4.5 and more negative values under native (unbuffered) and pH 7 conditions. The lower ZP observed at pH 4.5 shows reduced electrostatic repulsion between particles, promoting aggregation and resulting in the largest particle sizes for both beverage systems, as was observed in particle size results. In contrast, the more negative zeta potential values observed at native and pH 7 conditions increased electrostatic stabilization, limiting particle aggregation and producing substantially smaller particle sizes. This interpretation is consistent with the pH‐dependent changes in surface charge reported by (Chandrapala et al. 2012). These findings demonstrate a clear relationship between surface charge and particle size, highlighting the important role of electrostatic interactions in governing the colloidal stability of WPPC‐ and WPI RTM dispersions (Table 5). Previous studies have suggested that calcium and phosphate interactions contribute to protein aggregation and influence the physicochemical properties of whey systems (Chandrapala et al. 2012). These factors may also contribute to the pH‐dependent differences observed in the present study.

## 12 Microscopic Evaluation

Microscopy revealed that both WPPC and WPI RTM dispersions presented more heterogeneous microstructures at pH 4.5 than at pH 7 and native (Figure 6). The acidic dispersions contained larger and more irregular aggregates, indicating increased particle association under acidic conditions. In contrast, samples at pH 7 showed a more homogeneous distribution of smaller particles with fewer large aggregates, suggesting improved colloidal stability.

**FIGURE 6:**
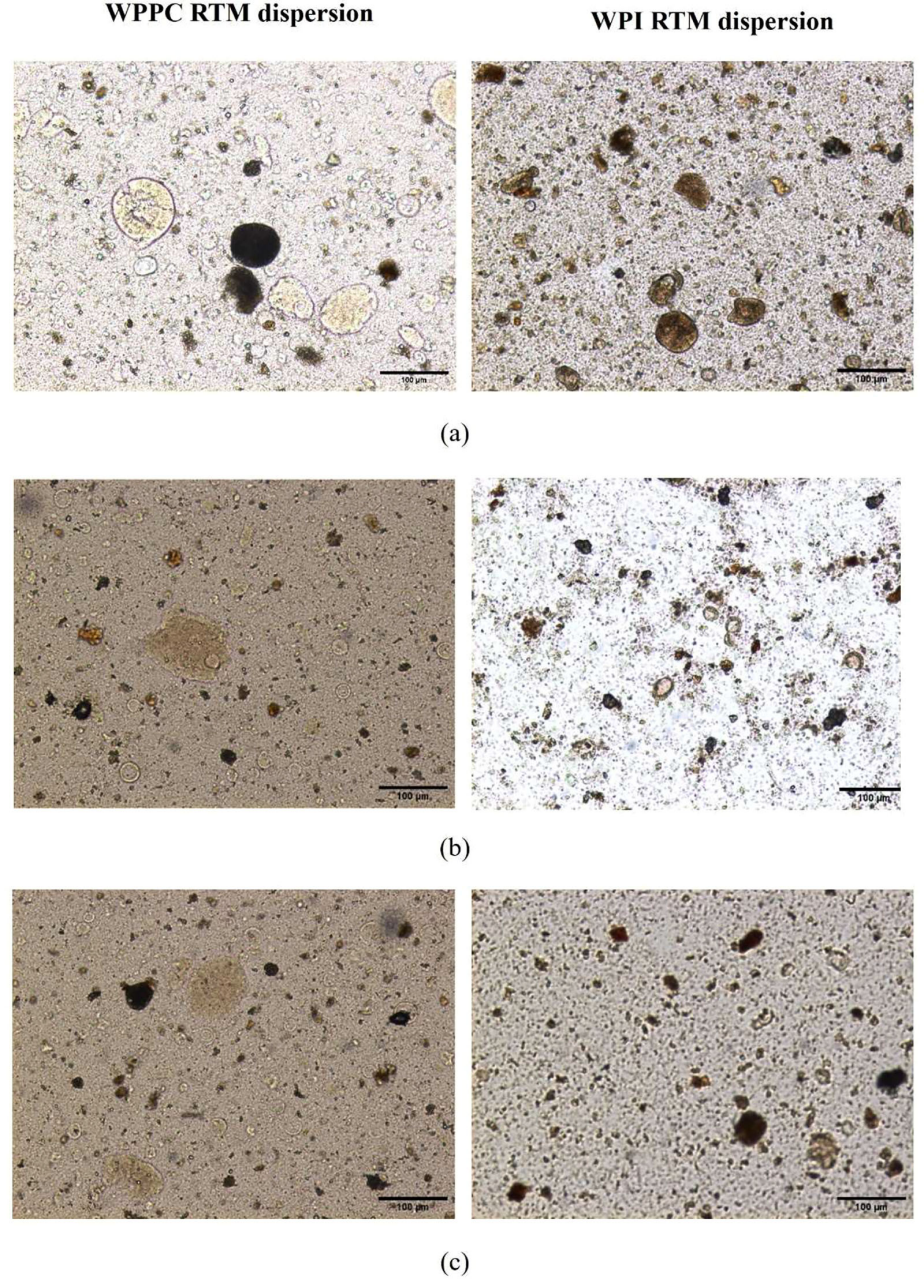
Representative light microscopy images of WPPC and WPI RTM dispersions at (a) pH 4.5, (b) native (unbuffered), and (c) pH 7. Images illustrate differences in particle aggregation and microstructure as influenced by pH. Scale bar = 100 µm.

The differences were observed between the two protein systems. The WPPC dispersions contained numerous large spherical particles, several of which presented an Eosin Y‐positive peripheral rim surrounding a lightly stained interior. Because Eosin Y preferentially stains proteins, the observed morphology may be consistent with protein‐rich interfacial layers surrounding lipid‐rich regions associated with the residual MFGM present in WPPC. However, as Eosin Y is not lipid‐specific, the composition of these structures cannot be confirmed from light microscopy alone.

In contrast, the WPI dispersions contained fewer large spherical structures and appeared more uniform, consistent with the substantially lower lipid and phospholipid content of commercial WPI. The microscopy observations were consistent with the particle size measurements, which showed significantly larger particles at pH 4.5 than under native and pH 7 conditions.

## 13 Rheological Evaluation

### 13.1 Apparent Viscosity

The rheological behavior of dispersions prepared from the WPPC RTM and the WPI RTM powder was evaluated at 10% (w/w) dispersion concentration and pH (7.0) to assess flow behavior relevant to beverage applications. These measurements were conducted on dispersions prepared in water. Apparent viscosity data for both systems were well described by the power‐law model (R^2^> 0.98 for all samples), indicating non‐Newtonian flow behavior.

Both systems exhibited shear‐thinning behavior, as indicated by flow behavior index (n) values less than 1. Shear‐thinning behavior is characteristic of whey protein dispersions and depends on protein concentration, pH, and ionic environment (Tang et al. 1993). Such behavior is desirable for beverage products because viscosity decreases during drinking while maintaining sufficient body at rest.

The power‐law model parameters showed that the WPI system had a substantially higher consistency coefficient (*K* = 89.04 Pa·s^n^) compared with the WPPC system (*K* = 26.45 Pa·s^n^), indicating greater resistance to flow under the tested condition. The higher consistency coefficient observed for the WPI system may be associated with compositional differences between the beverage systems, including differences in protein concentration or protein purity. However, the specific mechanisms responsible for these differences were not evaluated in the present study. Similar increases in apparent viscosity and consistency coefficient with increasing protein concentration and purity have been reported for whey protein systems at neutral pH (Tang et al. 1993).

In contrast, the WPPC system exhibited a lower flow behavior index (*n =* 0.23) compared with the WPI system (*n =* 0.58), indicating a stronger shear‐thinning response and a more rapid decrease in viscosity with increasing shear rate. This behavior may be associated with compositional differences between the two beverage systems, including differences in protein, lipid, and other formulation components. However, the specific mechanisms responsible for these differences were not investigated in the present study.

It should be noted that these rheological measurements reflect differences between two formulated RTM systems rather than purified protein ingredients, and therefore formulation components such as lipids and other ingredients may also contribute to the observed differences in flow behavior.

Despite exhibiting lower apparent viscosity, the WPPC dispersion maintained stable shear‐thinning behavior across the evaluated shear conditions. From a formulation perspective, the lower consistency coefficient and stronger shear‐thinning behavior of the WPPC system may support desirable drinking characteristics for protein beverage applications.

Overall, these results indicate that dispersions prepared from WPPC RTM powders exhibit flow behavior suitable for beverage formulations. Differences in viscosity and shear response were observed between the beverage systems; however, the specific contributions of individual formulation components were not investigated in the present study.

## 14 Sensory Evaluation

Consumer sensory evaluation was conducted to assess the acceptability of the reconstituted WPPC beverage system relative to the reconstituted commercial WPI beverage system, with results summarized in Table 3. Consumer liking was assessed using a 9‐point hedonic scale, a widely accepted method for evaluating food preference and acceptance (Drake 2007). The consumer panel (*n =* 100) consisted of 55% female and 45% male participants. With respect to age distribution, 46% of participants were between 18–24 years old, 33% were between 25–34 years old, 11% were between 35–44 years old, 6% were between 45–54 years old, and 4% were between 55–64 years old.

**TABLE 3:** Consumer sensory scores for the WPPC‐ and commercial WPI‐based ready‐to‐mix beverages (*n =* 100).

Attribute	WPPC‐based beverage	Commercial WPI‐based beverage	*P‐*value
Overall liking	5.51	6.36	0.0002*
Appearance	6.09	6.05	0.83
Texture	6.28	6.16	0.59
Aroma	5.99	6.46	0.01*
Flavor	5.07	6.06	0.001*
Aftertaste	4.94	5.47	0.001*
Color	6.06	6.50	0.03*
Purchase intent†	2.96	3.31	0.03*

*Note*: Values represent mean consumer scores (*n =* 100). P‐values were obtained using a linear mixed‐effects model with beverage formulation as a fixed effect and participant as a random effect. Sensory attributes (overall liking, appearance, texture, aroma, flavor, aftertaste, and color) were evaluated using a 9‐point hedonic scale (1 = dislike extremely; 9 = like extremely). †Purchase intent was evaluated using a 5‐point scale (1 = definitely would not buy; 5 = definitely would buy). *P*‐values indicate comparisons between beverage systems, with statistical significance defined as *p* < .

**TABLE 4:** Mean particle size of WPPC‐ and WPI‐based RTM dispersions at different pH conditions (µm).

	pH			
	4.5	Native (unbuffered)	7	P‐value
WPPC‐based RTM dispersion	1.18 ± 0.21^a^	0.51 ± 0.00^b^	0.59 ± 0.02^b^	< 0.03
Commercial WPI‐based RTM dispersion	1.99 ± 0.34^a^	0.81 ± 0.04^b^	0.80 ± 0.04^b^	< 0.01

*Note*: Values are presented as mean ± SEM (*n =* 3). Different superscript letters within the same row indicate significant differences (Tukey's test, *p* < 0.05).

**TABLE 5:** Zeta potential of WPPC‐ and WPI‐based RTM dispersions at pH 4.5, pH 7.0, and native (unbuffered) conditions.

	pH			
	4.5	Native (unbuffered)	7	*P*‐value
WPPC‐based RTM dispersion (mV)	−3.96 ± 0.70^a^	−11.01 ± 0.91^b^	−23.57 ± 1.21^c^	< 0.002
Commercial WPI‐based RTM dispersion (mV)	−2.84 ± 0.50^a^	−8.18 ± 0.38^b^	−23.90 ± 0.87^c^	< 0.002

*Note*: Values are presented as mean ± SEM (*n =* 3). Different superscript letters within the same row indicate significant differences (Tukey's test, *p* < 0.05).

Approximately 29% of participants indicated that they purchase protein‐based beverages several times per week, whereas 3% reported that they never purchase such products. The remaining participants reported occasional or infrequent protein‐based beverage purchasing, indicating a consumer panel with a range of experience levels relevant to assessing general consumer acceptance.

No significant differences were observed between the reconstituted WPPC and WPI beverage systems for acceptability of appearance and texture (Table 3). The lack of difference in texture perception is consistent with the rheological results. Although the WPPC system exhibited lower apparent viscosity than the WPI‐based system under pH (7) buffer conditions, it should be noted that sensory samples were prepared in milk, which differs in both pH and composition. Because viscosity is pH‐ and matrix‐dependent, direct comparison between instrumental viscosity and perceived texture should be interpreted with caution. Nevertheless, both systems exhibited stable shear‐thinning behavior consistent with beverage applications.

Significant differences were observed for flavor, aftertaste, overall liking, color, aroma, and purchase intent (*P* < 0.05; Table 3). Differences in the hedonic attributes ranged from approximately 0.4 to 1.0 points on the 9‐point hedonic scale. Purchase intent was evaluated separately using a 5‐point scale. This indicates moderate but perceptible differences in consumer preference. However, the two beverage systems differed in formulation composition, including levels of fat, salt, and sugar; therefore, differences in sensory scores cannot be attributed solely to the protein source. Rather, they should be interpreted as differences between two complete beverage formulations.

The commercial WPI beverage received significantly higher liking scores for overall liking, aroma, flavor, aftertaste, and color (*P* < 0.05), as well as a significantly higher purchase‐intent score (*P* < 0.05). No significant differences were observed for appearance or texture between the two beverages.

Although the commercial beverage received higher scores for several sensory attributes, the differences between formulations were generally less than one point on the 9‐point hedonic scale, suggesting moderate rather than substantial differences in consumer acceptance. As expected, the commercial beverage represents an optimized market‐ready formulation, whereas the WPPC beverage was developed as a prototype to evaluate the feasibility of incorporating WPPC into a ready‐to‐mix beverage system. Purchase intent did not differ significantly by gender for either beverage system (*P* > 0.05; Table S1). Among the age groups included in the statistical analysis (18–24, 25–34, and 35–44 years), no significant differences in purchase intent were observed for either beverage system (*P* > 0.05; Table S2). Participants aged 45–54 (*n =* 6) and 55–64 years (*n =* 4) were excluded from the age‐group statistical analysis because of the small sample sizes in these categories.

These findings highlight the importance of integrating instrumental measurements with consumer evaluation when assessing the suitability of novel dairy ingredients for beverage applications. In the present study, despite differences in instrumental viscosity and functional properties, no significant differences between the WPPC‐ and WPI‐based beverages were observed for appearance or texture.

While differences in composition of the beverage matrix may have contributed to sensory perception (Ammari and Schroen 2018), the specific mechanisms underlying the observed sensory differences were not evaluated in the present study. Additional studies using compositionally matched formulations would help isolate the contribution of WPPC to flavor, mouthfeel, and overall consumer acceptance.

## 15 Limitations

A limitation of the present study is that functional measurements were conducted in water dispersions rather than in the final milk‐based beverage matrix. Because milk contains caseins, milk fat, and minerals that may influence protein interactions and interfacial behavior, the functional properties reported here should be interpreted as standardized ingredient‐level measurements rather than direct predictors of performance in the final beverage system. Future studies evaluating WPPC directly in milk‐based systems would provide additional application‐relevant information.

An additional limitation is that the WPPC‐ and WPI‐based beverages were not compositionally matched and differed in ingredients such as fat, sugar, sodium, and total solids. Therefore, differences observed in functional and sensory properties should be interpreted as comparisons between two complete beverage formulations rather than effects attributable solely to the protein ingredient. Future studies using compositionally matched formulations are needed to isolate the specific contribution of WPPC.

Another limitation of the present study is that the WPPC RTM powder was produced from a single manufacturing batch. Therefore, batch‐to‐batch variability and manufacturing reproducibility could not be evaluated. Consequently, the functional, physicochemical, and sensory characteristics reported in this study represent only the batch evaluated. Future work should include multiple independently manufactured batches to assess manufacturing variability and the reproducibility of the observed results.

## 16 Conclusion

This study demonstrated the feasibility of incorporating WPPC into an RTM beverage system. WPPC exhibited pH‐dependent functional behavior consistent with whey‐derived ingredients, with improved functionality under neutral and native (unbuffered) conditions. Although the WPPC RTM powder showed lower water‐holding capacity and solubility than the commercial WPI RTM powder, it maintained adequate heat stability, emulsifying functionality, and shear‐thinning behavior suitable for beverage applications.

Sensory evaluation demonstrated that the commercial WPI beverage received significantly higher consumer liking scores for overall liking, aroma, flavor, aftertaste, and color, as well as a significantly higher purchase‐intent score, while no significant differences were observed for appearance or texture. The feasibility of incorporating WPPC into ready‐to‐mix (RTM) beverage formulations supports its potential as a functional dairy ingredient while highlighting opportunities to further improve flavor, aroma, and aftertaste to enhance consumer acceptance.

Overall, the results support the potential application of WPPC as a value‐added dairy coproduct for ready‐to‐mix beverage systems. Future studies using compositionally matched formulations and multiple production batches are warranted to further characterize the specific contribution of WPPC to beverage functionality and consumer acceptance.

## Author Contributions

**Fatemeh Jalil Mozhdehi**: methodology, formal analysis, writing – original draft, writing – review and editing, data curation, visualization. **Sheng‐Luen Shih**: data curation, writing – review and editing. **Susan G. Larson**: methodology. **Scott A. Rankin**: methodology, writing – review and editing. **Bradley W. Bolling**: writing – review and editing, funding acquisition, conceptualization. **Gulustan Ozturk**: methodology, writing – review and editing, funding acquisition, project administration, supervision, conceptualization, resources.

## Conflicts of Interest

The authors declare no conflicts of interest.

## Supporting information




**Supplementary Table**: jfds71536‐sup‐0001‐TableS1‐S2.docx
