# Supplementary material for: Functional, Rheological, and Sensory Properties of a Whey Protein Phospholipid Concentrate‐Based Ready‐to‐Mix Beverage System
Source: J Food Sci. 2026 Oct 9;91(10):e71536. doi: 10.1111/1750-3841.71536 (PMC13651926; doi:10.1111/1750-3841.71536)
Supplement: Supplementary file 1 [file JFDS-91-0-s001.docx]

**Supplementary Table S1. Effect of gender on purchase intent scores for the WPPC- and commercial WPI-based RTM beverages**

| **Beverage** | **Female** | **Male** | **P-value** |
| --- | --- | --- | --- |
| WPPC-based beverage | 3.57 | 3.24 | 0.238 |
| WPI-based beverage | 2.78 | 2.87 | 0.705 |

*Purchase intent was evaluated using a 5-point scale, where 1 = definitely would not buy and 5 = definitely would buy. Values represent mean purchase intent scores. No significant differences in purchase intent were observed between female and male participants for either beverage (P > 0.05).*

**Supplementary Table S2. Effect of age group on purchase intent scores for the WPPC- and commercial WPI-based RTM beverages**

| **Beverage** | **18–24 years**  **(n=46)** | **25–34 years (n=33)** | **35–44 years (n=11)** | **P-value** |
| --- | --- | --- | --- | --- |
| WPPC-based beverage | 3.31 | 3.54 | 3.70 | 0.517 |
| WPI-based beverage | 2.79 | 2.72 | 3.30 | 0.433 |

*Purchase intent was evaluated using a 5-point scale, where 1 = definitely would not buy and 5 = definitely would buy. Values represent mean purchase intent scores. Age-group comparisons included participants aged 18–24, 25–34, and 35–44 years. Participants aged 45–54 (n = 6) and 55–64 years (n = 4) were excluded from the statistical analysis because of the small sample sizes in these categories. No significant differences in purchase intent were observed among the age groups included in the analysis for either beverage (P > 0.05).*
